# Supplementary material for: Dynamics and resiliency of networks with concurrent cascading failure and self-healing
Source: PLoS One. 2022 Nov 15;17(11):e0277490. doi: 10.1371/journal.pone.0277490 (PMC9665362; doi:10.1371/journal.pone.0277490)
Supplement: Attachment — Submitted filename: Response to Reviewer #2.pdf [file pone.0277490.s001.pdf]

Response to Reviewer #2

Reviewer #2: This manuscript proposes a Dynamics and resiliency of networks with concurrent cascading failure and self-healing. The idea is good and theoretically verified; however, it needs to spot the light on the main contribution, and many issues should be addressed according to the following comments:

1- The "Abstract" section should be more intensively focused on the main idea directly and must contain the contribution of this manuscript with numerical result indicators.

Authors' response: *The authors believe that we have clearly described the main ideas, contributions, and results in the Abstract. Regarding the "numerical indicators", our opinion is that they might be less important than the algorithm and simulation framework developed and demonstrated in this study. The numerical results are from the simulations of two synthetic networks with 5000 nodes and 10000 links. The numerical indicators may be applicable only to these specific networks. This is why the main conclusions of this work does not include such numerical indicators.*

2-It is mandatory to add the figure of the system model.

Authors' response: *The authors do not fully understand what is a "figure of the system model". We assume that it might mean something like a block diagram of a control system. Our model is based on complex networks with 5000 nodes and 10000 links, the visualization of which is messy and more importantly carries little useful information. Therefore, instead of plotting the complex networks, we provided the details of how we generate the complex networks in Sec. 2.2. If the reviewer insists, we would like to point to the insets of Figure 2, which provides snapshots of the networked systems.*

The detailed description of the dynamic processes that occur in the complex networks can be found in Sec. 2.1. The title of Sec. 2.1 might be a little misleading. In the manuscript, we change it to "Dynamic processes on networks." The authors think that the description is clear and no diagram is needed.

check all the citing references of equations. In addition, check carefully all the abbreviation definitions, symbols, and standard units in the whole manuscript. I catch some errors and the other symbols were not defined.

Authors' response: *There is no labeled equation in the manuscript. All the notations are summarized and explained in Table I. We have made sure that there are no undefined symbols. Regarding "standard units," most of the quantities are dimensionless (e.g., ratios, number of active nodes, etc.). The time is measured by discrete time-steps and is essentially dimensionless. The load and capacity of a node are simply some numerical values for comparison with each other; their absolute values and units are not essential in the abstract networked systems under study.*

3- The resolution and quality of all result figures should be modified; they should be presented as close to the camera-ready format. Also, please don't use the symbol abbreviations on X-Y-axes, they must have the full name with their units. Further, a lot of figures with poor quality X-Y axes, it must write by zooming in on the effective zones.

Authors' response: *In the original manuscript, the resolution of all figures is adequate. Upon acceptance, we will submit high-resolution images for each figure.*

4- Table of comparison need to be addresses in various terms to highlight the performance of conducted work and related works in literature.

Authors' response: *We would like to take the opportunity to clarify that our work is not aimed at superior performance compared to previous works. As explained in the Introduction, our main innovation is to study the resilience of networked systems with concurrent cascading failure and self healing. Our work delivered new results to reveal the dependence of system resiliency on various model parameters.*

Finally, the authors would like appreciate the reviewer's effort to help improve the manuscript.
